# Lymphopenia during ^177^Lu-DOTATATE therapy leading to recurrence of tuberculosis: a case report

**DOI:** 10.1186/s41824-022-00157-y

**Published:** 2022-12-13

**Authors:** Sarah Boughdad, Michael Da Mota, Mélanie Mendes De Carvalho, Maria Firsova, John O. Prior, Niklaus Schaefer

**Affiliations:** Department of Nuclear Medicine and Molecular Imaging, Lausanne University Hospital, Lausanne, Switzerland

**Keywords:** ^177^Lu-DOTATATE, Pancreatic NET, Lymphopenia, Tuberculosis

## Abstract

We report the case of a 72-year-old woman who presented with tuberculous arthritis during the setting of ^177^Lu-DOTATATE therapy for a grade-2 neuro-endocrine pancreatic tumor with liver metastases. We hypothesized that this recurrence might have been related to the occurrence of lymphopenia, which is common during PRRT. Indeed, though lymphopenia is frequently dismissed, it could lead to the development of opportunistic diseases and its severity should be examined, especially in case of abnormal clinical symptoms.

**Supplementary Information:**

The online version contains supplementary material available at 10.1186/s41824-022-00157-y.

## Introduction

Peptide receptor radionuclide therapy (PRRT) in NET has shown good results in metastatic patients with grade-1 or grade-2 tumor originating from the midgut (Oronsky et al. 2017; Mortenson and Bold 2002; Anderson and Bennett 2016). PRRT targeting somatostatin receptor especially SSTR2 demonstrated increased objective response rate, overall survival and quality of life (Bodei et al. 2015). In NET patients with primary pancreatic tumors, PRRT showed promising results in non-randomized studies, and though phase III results are pending (NCT02489604), it is often recommended as a second-line treatment with rare adverse events (Strosberg et al. 2015, 2018). Lymphopenia is frequently reported during PRRT, with transient lymphopenia reported in 18% of patients in the NETTER-1 trial without further impact on the course of PRRT (Marinova et al. 2018; Strosberg et al. 2017). Yet, the occurrence of unusual clinical symptoms in PRRT patients presenting with acute or severe lymphopenia should not be systematically dismissed as insignificant, as it might be lead to an underlying infectious disease.

## Material and methods

We reported the case of a 72-year-old woman for whom a history of primary tuberculosis infection during childhood was unclear and who presented with tuberculous arthritis during the setting of ^177^Lu-DOTATATE PRRT. Initially, this patient had abdominal pain at diagnosis and a contrast-enhanced computer tomography (CE-CT) lead to the diagnosis of primary pancreatic NET with synchronous liver metastases (grade 2 and Ki-67 15% at biopsy). The patient received a first-line treatment using somatostatin analogues without significant tumor response or improvement of the clinical symptomatology. A second and third line of treatment using chemotherapy (gemcitabine/oxaliplatin followed by capecitabine/temozolomide) only stabilized the disease for less than a year. Indeed, a follow-up FDG PET/CT scan showed a mild metabolic progression in both primary pancreatic tumor and liver metastases. ^68^ Ga-DOTATATE PET/CT showed high uptake in both primary pancreatic tumor and liver metastases (Additional file 1: Fig. S1 **)**. Therefore, in agreement with the standard of care and after approval of the dedicated multidisciplinary board, initiation of PRRT using ^177^Lu-DOTATATE was decided. Patients or the public were not involved in the design, or conduct, or reporting, or dissemination plans of this case report.

### PRRT Therapy

**Fig. 1:**
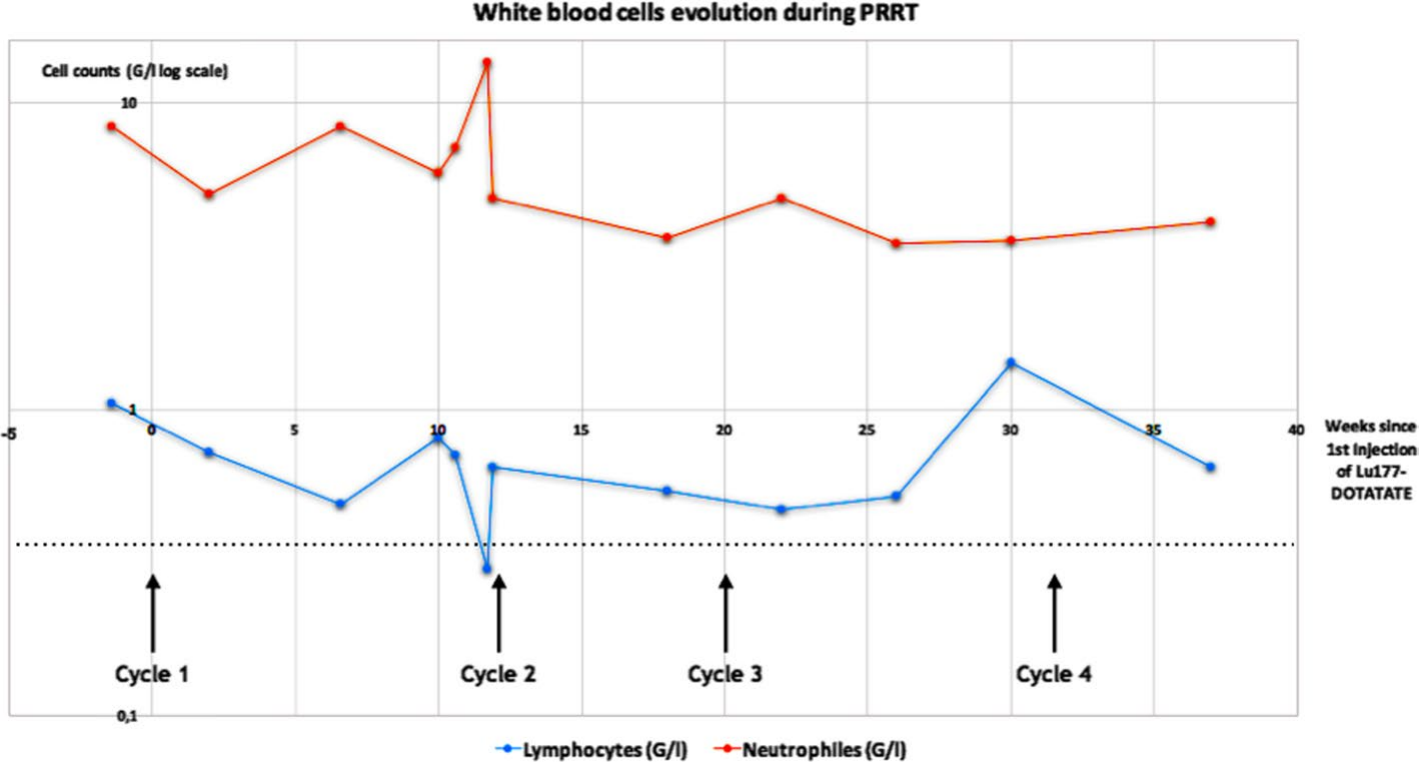
Changes in white blood cell counts during PRRT (lymphocytes (blue) and neutrophils (orange); logarithmic scale); dotted line: definition of severe lymphopenia (value < 0.5G/l); * in red: values for CRP (mg/ml)

The patient received the first cycle (C1) of PRRT with a reduced activity of 5.6 GBq instead of standard 7.4 GBq due to her low body mass index (18 kg/m^2^) though clinical examination was unremarkable and the blood work was within normal range (Fig. 1). Between the first and second cycle, the patient reported a traumatic fall involving the right elbow and the right ankle with persistent inflammatory pain in the latter. At clinical examination before the second treatment, there was a limping of the right ankle with pain at mobilization and swelling, but no redness nor fever was found. In light of those symptoms, PRRT was postponed until further investigations. Same day blood work showed elevated white cell counts with 13.5 (N > 1.8) G/L for neutrophils (NP), whereas lymphocytes (LP) were below 0.4 (N > 1.5) G/L (Fig. 1). In addition, C-reactive protein (CRP) was elevated at 102 (N < 5) mg/L, but procalcitonin was normal at 0.08 (N < 0.5) µg/L) and blood cultures were negative. Similarly, synovial fluid withdrawal did not show any inflammatory nor cancerous cells nor crystals, and standard ankle and chest X-rays were also negative (Additional file 1: Fig. S2). Thus, the resulting diagnostic was a mono-arthritis of undefined origin ranging from paraneoplastic, chondrocalcinosis, inflammatory rheumatism to insidious infection. Additionally, as the blood work normalized the next day without any treatment (NP: 4.9 G/L and LP: 0.65 G/L, Fig. 1) and in the absence of conclusive clinical sign of active infection, the patient received the second cycle of PRRT with a further reduced activity of 3.8 GBq of ^177^Lu-DOTATATE with good clinical tolerance. Clinical assessment before injection of the third injection of ^177^Lu-DOTATATE was normal, and the patient did not present any pain at the clinical examination of her right ankle. Between the 3rd and 4th cycle of PRRT, the patient was again investigated for the same pain of the right ankle, which was explored without conclusive results leading to a delay in initiating the 4th injection of ^177^Lu-DOTATATE by 4 weeks. At the time of C4,  the blood work was satisfactory though a slight Lymphopenia was seen 1.4 G/l (Fig. 1), and the treatment was carried out with injection of  half the standard activity  of ^177^Lu-DOTATATE with a good clinical tolerance.

**Fig. 2:**
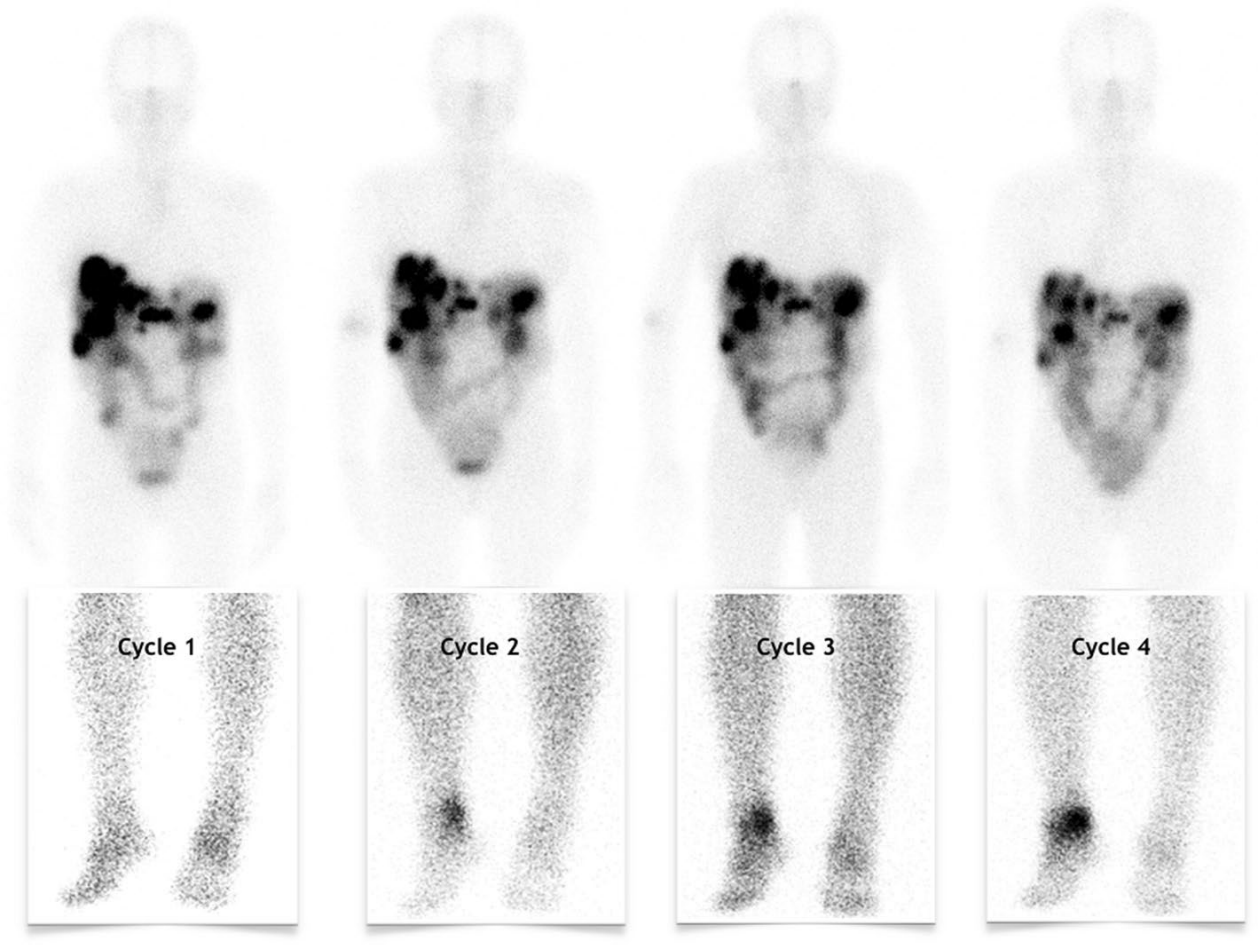
Whole body planar scintigraphy images in the anterior view 2-days post PRRT with zoom images below showing increasing uptake in the right ankle from cycle 2 to 4

**Fig. 3:**
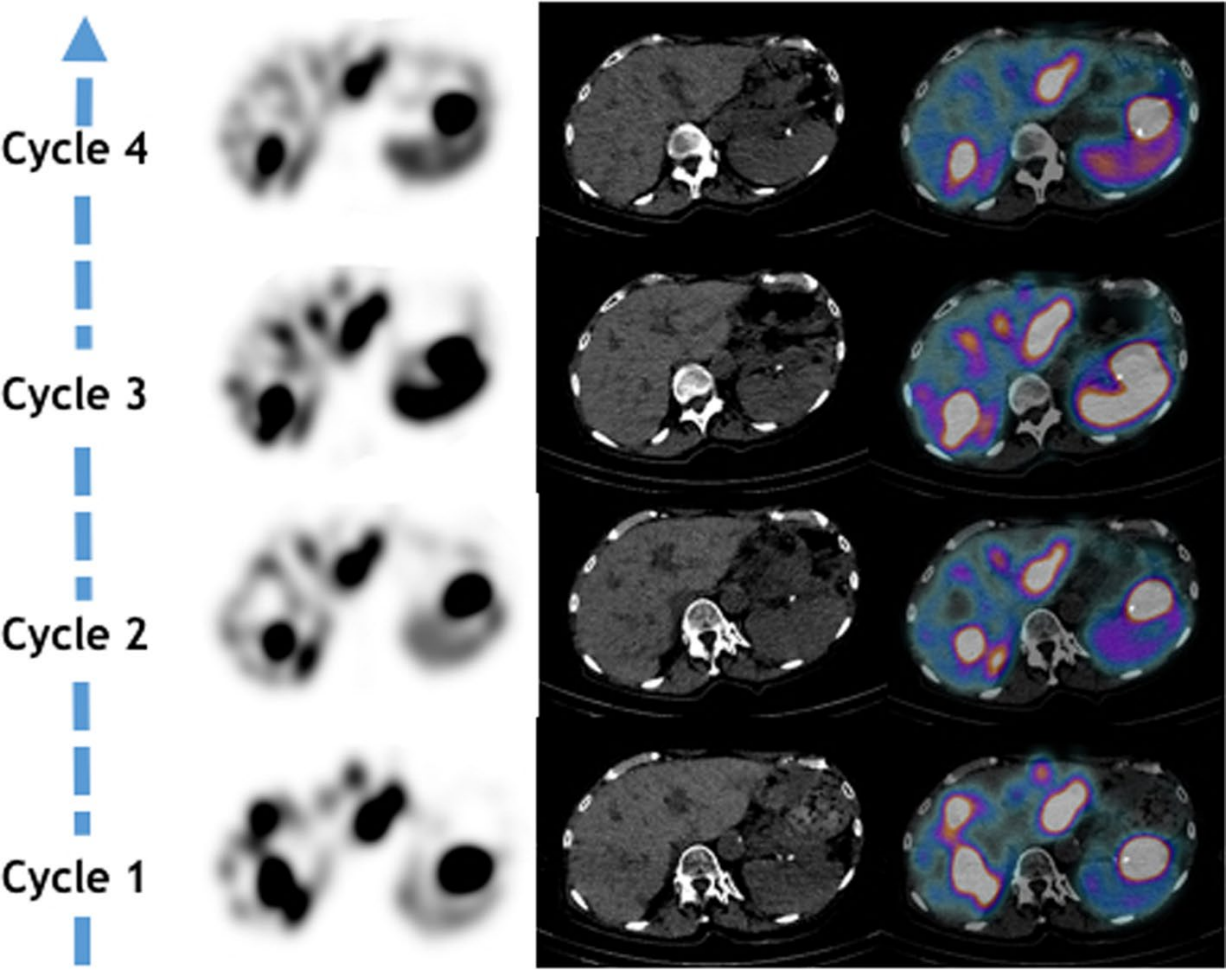
Axial slices of SPECT, CT and fused SPECT/CT images showing uptake in the primary pancreatic tumor and liver metastases after each cycle of PRRT

Interestingly, though all investigations remained negative during the course of PRRT, post-injection planar scintigraphy at 48 h showed from the second cycle an increasing mild uptake of the right angle (Fig. 2). In contrast, on additional SPECT/CT imaging, there was a continued decrease in Lu177-DOTATATE uptake in NET lesions, particularly in the pancreatic tumor and liver metastases (Fig. 3). Ultimately, the patient presented with a good response on the ^68^ Ga-DOTATATE PET/CT scan done 4 months after the end of PRRT (Additional file 1: Fig. S1).

However, two months after the end of PRRT, the patient experienced once again an acute pain in the right ankle. Blood tests, ankle ultrasound and CT scan were suspicious for an active infection, with the latter showing bone erosions in favor of an aggressive process. The patient underwent an arthroscopy with multiple biopsies, an arthrotomy of the medial malleolus and a curettage of the infection site. Biopsies and subsequent bacterial cultures led to retain the diagnosis of a chronic tuberculosis infection with mycobacteria tuberculosis of the right ankle. As tuberculosis was suspected, a thoracic CT scan was performed and did not show any pulmonary sign of the disease***. ***After a pneumology referral, a long-term anti-tuberculosis treatment using the four-drug fixed-dose combination regimen RIMSTAR® (9), combined with a B6 vitamin substitution, was started.

## Discussion

To the best of our knowledge, this is the first study reporting the likely recurrence of mycobacteria tuberculosis during the course of PRRT in patients with NET. This finding is interesting because adverse events or abnormal non-tumoral uptakes of ^177^Lu –DOTATATE are uncommon (Strosberg et al. 2015, 2018). Indeed, lymphopenia was frequent and has been reported in 18% of the patients in NETTER-1 trial but it was usually transient and of no consequence during the course of PRRT (Strosberg et al. 2017). Most common adverse events in the NETTER-1 trial were gastro-intestinal disorders, fatigue, musculoskeletal pain followed by blood disorders mainly thrombocytopenia (25%), lymphopenia (18%) and anemia (14%), which were mainly moderate (Strosberg et al. 2017). Thus, despite post-injection planar scintigraphy showing an increasing uptake of the right ankle for this patient from C2 to C4, this uptake was considered to be a false-positive related to local inflammation (Fig. 2). Especially, since all SPECT/CT images done after cycle 2 to 4, all showed a decrease in NET lesions uptake suggestive of an early response to PRRT which was not coherent with the appearance of new secondary bone locations (Fig. 3). Additionally, the patient had a history of traumatic fall between C1 and C2 and all investigations remained negative for an active infection. Indeed, it has been reported in the literature that inflammatory cells such as lymphocytes and macrophages also expressed SSTR2, which was in our opinion coherent with a reactive process. Nonetheless, the patient did present with an acute and severe lymphopenia during the setting of PRRT, which is often underestimated even so it showed the highest level of grade 3 and 4 events (9%) in the NETTER-1 trial (8, 10). Indeed, its presence does not currently require the nuclear physician to postpone or reduce the activity of ^177^Lu-DOTATATE to be injected. However, it has been demonstrated in the literature that acute and severe lymphopenia could lead to the recurrence or occurrence of opportunistic disease in immunosuppressed patients mainly transplants or HIV patients but also impact outcomes in patients with sepsis especially with LP < 0.5G/L (Fuehner et al. 2019). Thus, when we looked retrospectively at the patient history that is the concomitant right ankle pain with an episode of severe lymphopenia and the abnormal uptake on the post-treatment planar scintigraphy, we hypothesized that it could have been associated with the recurrence of this tubercular disease. Besides, *mycobacteria tuberculosis* is not a rare pathogen and even so patients could not present with a clear history of primary infection and it should be considered in front of abnormal or unexplained clinical symptoms associated with lymphopenia (Fuehner et al. 2019; Kaul and Chauhan 2014; Sreejith et al. 2010; Cock et al. 1995). Interestingly, except for the blood work done prior to PRRT initiation and the one before the last cycle (Fig. 1), the patient always presented with at least moderate lymphopenia raising the question of systematic screening for latent tuberculosis in at-risk patients before PRRT initiation (Vries et al. 2014). This might be particularly interesting in elderly patients with an inconclusive history or those in a precarious situation especially for patients with borderline LP counts at PRRT initiation and/or were previously treated with potentially hematotoxic treatments. Indeed, post-treatment lymphopenia has been reported in various solid tumors in patients treated with chemotherapy or radiation therapy (Wang et al. 2021; Campian et al. 2013). In this case, the patient was previously treated with 2 lines of chemotherapy and there was a tendency to mild lymphopenia with a lymphocyte cells count of 1.2 G/l seen 2 months before the start of PRRT while neutrophils, hemoglobinemia and platelets were all within normal range. The presence of local inflammation following the traumatic fall reported by the patient may have been an additional relevant factor leading to the recurrence of tuberculosis at this particular site.

Thus, our report showed that lymphopenia might not be an insignificant blood finding and could require further investigations, especially in front of abnormal clinical symptoms during the course of PRRT. This is all the more important that severe lymphopenia could occur at reduced dose as for this patient, who received a dose lower than the current fixed dose of 7.4 GBq recommended in the literature (Bodei et al. 2015), (Strosberg et al. 2015, 2018)

## Conclusion

To resume, though lymphopenia is frequent and usually benign during the course of PRRT it could be associated with opportunistic diseases and its severity should be examined especially in case of abnormal clinical symptoms.

## Supplementary Information

**Additional file 1: Fig. S1.** ^68^Ga-DOTATATE PET/CT image (SUV scale: 0–12.5 g/mL): MIP images **a** and **e** before PRRT and after PRRT; **b**), **c**) and **d**) axial slices CT, Fused PET/CT and CE-CT (Top images=after PRRT). **Fig. S2**. Chest (**a**) and right ankle X-rays (front (**b**) and side (**c**)) before second injection of ^177^Lu-DOTATATE.

## Data Availability

Data and material can be made available upon request.

## Abbreviations

NET: Neuro-endocrine tumor
PRRT: Peptide receptor radionuclide therapy
SSTR2: Somatostatin receptor 2
CE-CT: Contrast-enhanced computer tomography
CRP: C reactive protein
C: Cycle
NP: Neutrophils
LP: Lymphocytes
