# Supplementary material for: Lymphopenia during 177Lu-DOTATATE therapy leading to recurrence of tuberculosis: a case report
Source: Eur J Hybrid Imaging. 2022 Dec 13;6:36. doi: 10.1186/s41824-022-00157-y (PMC9744994; doi:10.1186/s41824-022-00157-y)

**Supplemental figures:**

**Figure 1.** ^68^Ga-DOTATATE PET/CT image (SUV scale: 0–12.5 g/mL): MIP images a and e before PRRT and after PRRT; b), c) and d) axial slices CT, Fused PET/CT and CE-CT (Top images=after PRRT).


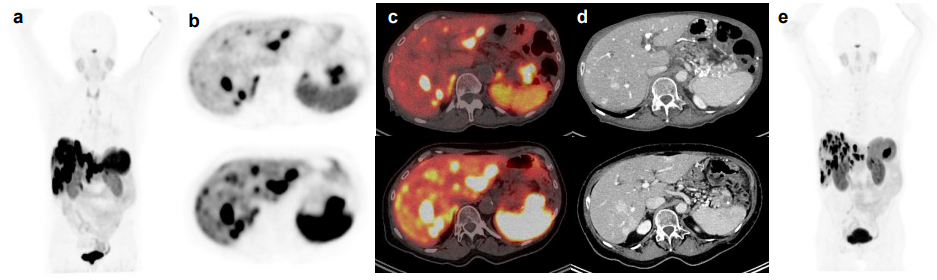


**Figure 2**. Chest (**a**) and right ankle X-rays (front (**b**) and side (**c**)) before second injection of ^177^Lu-DOTATATE.


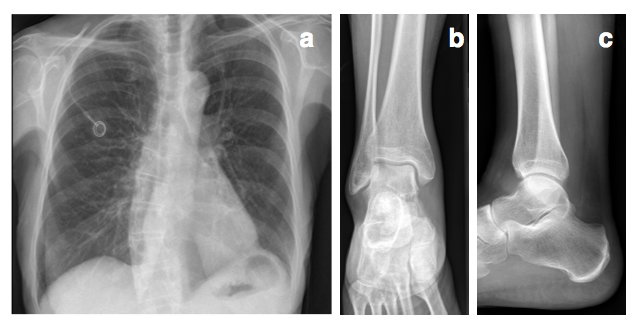

Supplement: Supplementary file 1 — Additional file 1: Fig. S1. 68Ga-DOTATATE PET/CT image (SUV scale: 0–12.5 g/mL): MIP images a and e before PRRT and after PRRT; b), c) and d) axial slices CT, Fused PET/CT and CE-CT (Top images=after PRRT). Fig. S2. Chest (a) and right ankle X-rays (front (b) and side (c)) before second injection of 177Lu-DOTATATE. [file 41824_2022_157_MOESM1_ESM.docx]
